# Agenda Setting and Evidence in Maternal Health: Connecting Research and Policy in Timor-Leste

**DOI:** 10.3389/fpubh.2015.00212

**Published:** 2015-09-10

**Authors:** Kayli Wild, Paul Kelly, Lesley Barclay, Nelson Martins

**Affiliations:** ^1^Institute for Human Security and Social Change, La Trobe University, Melbourne, VIC, Australia; ^2^ACT Health, ACT Government and Australian National University Medical School, Canberra, ACT, Australia; ^3^University Centre for Rural Health, University of Sydney, Lismore, NSW, Australia; ^4^Faculty of Public Health, Ministry of Health, Universidade da Paz, Dili, Timor-Leste

**Keywords:** Timor-Leste, evidence-based policy, maternity waiting homes, maternal health policy, policy theory, actors, agenda setting, power

## Abstract

The evidence-based policy (EBP) movement has received significant attention in the scientific literature; however, there is still very little empirical research to provide insight into how policy decisions are made and how evidence is used. The lack of research on this topic in low- and middle-income countries is of particular note. We examine the maternity waiting home policy in Timor-Leste to understand the role of context, policy characteristics, individual actors, and how evidence is used to influence the policy agenda. The research tracked the maternity waiting home policy from 2005 to 2009 and is based on in-depth interviews with 31 senior policy-makers, department managers, non-government organization representatives, and United Nations advisors. It is also informed by direct observation, attendance at meetings and workshops, and analysis of policy documents. The findings from this ethnographic case study demonstrate that although the post-conflict context opened up space for new policy ideas senior Ministry of Health officials rather than donors had the most power in setting the policy agenda. Maternity waiting homes were appealing because they were a visible, non-controversial, and logical solution to the problem of accessing maternal health services. Evidence was used in a variety of ways, from supporting pre-determined agendas to informing new policy directions. In the pursuit of EBP, we conclude that the power of research to inform policy lies in its timeliness and relevance, and is facilitated by the connection between researchers and policy-makers.

## Introduction

In order to improve public health outcomes, the need for evidence-based policy (EBP) has gained prominence over the last two decades. EBP promotes a rational and coordinated approach to policy-making based on a comprehensive assessment of research to build an evidence base (1). In this sense, evidence is the culmination of knowledge based on rigorous scientific research. Despite widespread advocacy of this approach, significant barriers to the use of both research and evidence remain. Oliver et al. (2) compared three systematic reviews on EBP and found the common barriers to using research across all three reviews were:

1. lack of personal relationships and contacts between decision-makers and researchers;
2. the need for research to be clearly and accessibly presented.

Similarly, Behague et al. (3) (p. 1542) found the EBP movement “has failed to curb long-standing problems with … advocacy-driven ‘fad-like’ shifts in policy recommendations.” The EBP literature has tended to focus on how research can have an impact on policy, rather than the policy process itself. Importantly, Oliver et al. (2) point to the lack of ethnographic and anthropological approaches to policy analysis. We contribute to filling this gap in knowledge by providing an ethnographic case study of how research and policy interact in a developing country health system. An in-depth analysis of the maternity waiting home policy in Timor-Leste helps to illuminate the complex processes behind policy decisions and the use of evidence in post-conflict and low-resource settings.

### Country context

Timor-Leste achieved independence from Indonesia following a violent referendum in 1999. Like other countries with a history of conflict, Timor-Leste has an extremely high maternal mortality ratio, estimated to be as high as 929/100,000 live births (4). Reducing maternal mortality and improving access to health facilities was prioritized by the Timorese government and the many non-government organizations (NGOs) and United Nations (UN) agencies entering the country during the reconstruction phase. Although there are many ways (5), the Ministry of Health in Timor-Leste, like many countries, chose to focus on promoting facility-based delivery (6–8). In order to improve access to facility-based birth for women in remote areas, the maternity waiting home idea was introduced by UNFPA representatives (9) and a National Maternity Waiting Home Strategy was developed by the Timorese Government in 2005 (10). The concept of maternity waiting homes was first developed by medical doctors in Africa in the 1950s (11). They are houses, located near hospitals, where women can wait in the weeks prior to their due date in order to give birth in a health facility (12). The concept has been promoted as a strategy to “bridge the geographical gap,” and thus aims to improve access to obstetric services for women in rural and remote areas (12) (p. 1). However, there is limited evidence to suggest they are an effective strategy in reducing maternal or perinatal mortality, or improving access to care for women in remote areas (13, 14).

The initial plan in Timor-Leste was to pilot maternity waiting homes in three districts, and this soon increased to five districts (15). Enthusiasm for the strategy grew at both the national and district level, and policy-makers suggested they should be established nation-wide. In 2007, they were incorporated into the national Basic Services Package, which meant that they could be implemented in every health post, health center, and hospital in the country (7). This enthusiasm for maternity waiting homes in Timor-Leste raises important questions about the use of evidence, why certain ideas are enthusiastically taken up at the local level, and the interests and agendas influencing policy decisions.

Some research has been conducted on global-level forces that influence national policy systems, but less is known about the mechanisms through which local actors influence policy, particularly in low-income countries (16). Behague et al. (3) point out that a deeper analysis of national and sub-national policy processes should be conducted to understand the factors which inhibit the use of evidence in policy. There has been a call for further research which incorporates the voices of local actors and assesses the weight of different causal factors and their interactive effects in raising issues on the political agenda (17). de Leeuw et al. (18) also argue that studying health policy requires an understanding of its development process. As researchers working in the country, we were in an ideal position to study the development and implementation of the maternity waiting home policy from 2005 to 2009.

### Theoretical orientation

In their review, Oliver et al. (2) argue that much of the research on EBP remains atheoretical and call for more critically and theoretically informed studies of decision-making. Drawing on policy theory from the political sciences provides a powerful tool kit to help explain *why* things are happening, beyond a description *that* they are happening (18).

Kingdon’s (19) work on how policies become prominent in national agendas has been one of the most influential in the discipline of policy analysis. Kingdon outlines how problems, policies, and politics merge at certain points to create windows of opportunity for governments to act. He highlights the importance of policy champions who act as advocates for policies that promote their values and personal interests, and protect their bureaucratic space. Kingdon’s theory is well articulated; however, the research was based in the United States in the 1970s and may not be applicable in the context of political processes in low-income countries where overseas aid features heavily.

Walt and Gilson (20) also emphasize the central role of actors and how their position, power, values, and expectations influence the policy process. Other significant factors in this model include context, policy content, and political processes. Importantly, policy analysis benefits from an understanding of the complexities and interrelationships of all these factors. Shiffman and Smith (17) have presented a similar framework for assessing political priority for global health initiatives, with a focus on actors, ideas, context, and issue characteristics.

Each model has considerable explanatory power. Common elements in these models point to the critical role of context, policy characteristics, and actors in influencing policy processes. What remains is to test the applicability of these elements in developing country health systems. If these models hold, then what is the role of evidence and how is it used to influence the policy agenda? This research uses the common elements of these models to assess the relative importance of political context, policy characteristics, international advisors, national actors, and different forms of evidence in prioritizing maternity waiting homes in Timor-Leste.

## Materials and Methods

This ethnographic case study of the maternity waiting home policy in Timor-Leste was carried out by the lead author, Kayli Wild, from 2005 to 2009 as part of her doctoral research (21). Kayli Wild conducted all interviews and was responsible for data collection and analysis. The co-authors Lesley Barclay, Paul Kelly, and Nelson Martins in their capacity as supervisors, provided input into study design, accessing key informants, and critically revising the manuscript. To position the researchers, Kayli Wild and Lesley Barclay could be considered feminist researchers with an interest in advocating for women’s preferences when shaping maternal health policy and services. At the outset, we took a critical approach to understanding the role of maternity waiting homes. Given the lack of evidence for the effectiveness of maternity waiting homes in the international literature, we wanted to use the most rigorous methods to test whether they were working to address the needs of rural women in Timor-Leste (14). The literature on EBP does not usually involve policy-makers as co-authors, indicating that they are largely absent in developing or carrying out policy-relevant research (2). During data collection for this research, co-author Nelson Martins became the Minister for Health in Timor-Leste. Thus, he acted as supervisor, participant observer, and the subject of research. We acknowledge the potential conflict of interest in this dual role and also the unique insights it can provide. Kayli Wild, as an independent researcher, has retained control of data analysis and interpretation. While we have protected the anonymity of specific policy actors we have remained candid about the role of senior decision-makers in the policy process.

Participants who were knowledgeable about maternal health policy were recruited in Dili as this is the capital city and central hub of decision-making and influence in Timor-Leste. Data were triangulated by interviewing informants from a range of organizations and from different levels within organizations, including local and international NGOs, UN agency representatives involved in maternal health, technical advisors, senior decision-makers in the Ministry of Health, and managers in the Maternal and Child Health and Policy and Planning departments (Table 1). Purposive snowball sampling was used whereby an initial small group of people involved with maternal health were identified and they were asked to recommend others who were knowledgeable about the maternity waiting home policy. Thirty-one interviews were conducted from July to December 2007. In addition to key informant interviews over 6 months of fieldwork, data were collected during regular visits to the country from 2005 to 2009. Other data collection methods included participant observation and informal discussion, attendance at meetings and workshops, and analysis of policy documents, workshop minutes, and NGO reports.

**Table 1:** **Number of interviews with key informants, by organization**.

Organization	Number of Interviews
Ministry of Health	9
UN Agency	9
International NGO	8
Local NGO	5
Total	31

A semi-structured interview schedule was used to interview key informants. This contained a set of open-ended questions relating to when and how the maternity waiting home concept first appeared in Timor-Leste, the role of different actors in influencing the policy agenda, the informants’ perspective on the maternity waiting home policy and its implementation, the use of evidence in policy decisions, and priorities in maternal health. Most interviews took place in person at the participant’s office in Dili, with the exception of one interview which was done over the telephone. As some important informants had left the country, effort was made to contact these people and correspond via email. Interviews were conducted in English when the participant was confident speaking the language, which was the case for 26 interviews. Five interviews were in Tetum using an interpreter, and these were subsequently translated and analyzed in English.

A plain language information sheet summarizing the research project was given to participants in English and Tetum. Signed consent to participate in the interview was gained from each participant, and permission was requested to use a digital tape recorder. Seventeen interviews were tape recorded and transcribed and for 14 interviews detailed notes were taken. Interviews usually lasted 1 h. The research was carried out with written permission from the Timorese Ministry of Health. Full ethical approval was obtained from the Human Research Ethics Committee at Menzies School of Health Research, Australia.

The qualitative research software NVivo7© was used to organize all data including transcripts, discussion notes, and field notes. Data analysis began with broad categories of evidence, actors, policy characteristics, and context. These categories were then populated by themes that emerged through coding each interview. Common and repeated themes were investigated across interviews to understand what informants were saying in relation to specific topics, and to assess the strength and importance of various themes. A comparative analysis was conducted to understand the views of internal/national versus external/international actors, as well as senior decision-makers versus mid-level department managers.

## Results

### Context

The crisis situation in Timor-Leste after the 1999 referendum resulted in many NGOs and UN agencies entering the country, each of whom brought new ideas, agendas, and resources. The influence of international organizations and global policy ideas was amplified because there was very little existing policy or bureaucratic structure. In the violent aftermath of the referendum, the Indonesian military and pro-integration militia destroyed much of the country’s infrastructure and most of the Indonesian health workforce and management left Timor-Leste. Thus, the policy space for health issues was expanded by this new layer of providers, ideologies, and ways of working. New policy options were introduced through UN agency representatives and the East Timorese Health Professionals Working Group (which was formed by the Timorese doctors who remained in the country and which became the foundation for the first Timorese Ministry of Health). Addressing the high rates of maternal mortality was firmly entrenched in Timor-Leste’s health policy agenda from the beginning. The crisis situation and incoming actors created a “window of opportunity” to take action and develop programs and policies.

### Policy characteristics

The National Maternity Waiting Home Strategy was developed by the Maternal and Child Health department, who were assisted by UNFPA and a technical working group (10, 15). Within the policy there was recognition of the need for other components of the health systems, such as emergency obstetric care and training midwives (6, 15); however, key informants described how maternity waiting homes came to be thought of as the *solution* for improving access to health facilities for women in remote areas.

People had kind of read about it and thought this will be the solution to East Timor’s problems because they have trouble getting to the facilities … There was an incredible momentum, and this feeling of ‘yes, that is the answer to our problem’. – Manager, international NGO, Dili

The prominence of maternity waiting homes was evident in most interviews. The popularity of the concept was demonstrated by the Ministry of Health holding a series of high-level policy workshops (one attended by more than 100 people) and the fact that maternity waiting homes had their own policy documents (10, 15). Enthusiasm for maternity waiting homes spanned all levels of the Ministry of Health, from senior decisions-makers to the department of Policy and Planning to district level health services (10). This meant that maternity waiting homes became rapidly and firmly entrenched on the government’s agenda.

The characteristics of the concept itself meant that it was an attractive policy option. First, it was seen to be a *logical* solution to the problem of access to facility-based birthing services for women living in remote areas.

I mean this is clearly one country where you would think of that type of maternal waiting homes. It offers itself quite naturally and logically. – Advisor 2, UN agency, Dili

In addition, maternity waiting homes fit comfortably within the medical model and the broader mandate of having all women birth in a health facility. Maternity waiting homes were also a safe policy option for politicians to promote, particularly over more controversial issues, such as family planning. Family planning was highly contentious in Timor-Leste because of society’s strong Catholic values, the history of enforced contraception during the Indonesian occupation, and the value placed on high fertility in the pursuit of nation building.

My take is that the Ministry can get very passionate about non-controversial things … [Family planning] is really the issue here, but it’s too controversial so I think there is a tendency to go with something that is a bit neutral, maternity waiting homes being one of those. – Program officer, UN agency, Dili

Another characteristic of maternity waiting homes was that they were visible structures through which government action could be demonstrated to the community. This was particularly relevant given the widespread destruction of infrastructure by the Indonesian military after the 1999 referendum. They were also seen as a way of involving the community and bridging the gap between district health services and outlying villages (15). This investment in infrastructure meant maternity waiting homes were an attractive option not only for national policy-makers but also for district health managers and midwives.

I think it felt very attractive, an idea that was very attractive and a possibility that it was not purely technical. It would probably mean the community is doing something for themselves, so in that sense a very strong political dimension. I mean, I don’t mean party politics as such but I mean reaching out, connecting with the community. – Advisor 2, UN agency, Dili

Once the national strategy was developed, The Ministry of Health began socializing the idea to the districts by convening a series of community meetings (10, 15). They were initially going to be piloted in three districts, but this was increased to four, then five districts. Then, in one dramatic up-scaling as part of the Basic Services Package, they were proposed for every one of the 65 sub-districts in the country (7). This represented a significant investment, with initial construction and equipment being US$40,000–60,000 and monthly running costs of US$2,745 (14, 15). The costs in the pilot districts were initially met by UNFPA and other local and international NGOs with the expectation the government would take over management and monthly expenses.

In the end, only two of the original pilot projects were implemented. Both failed to improve access for women in remote areas (14). Nevertheless, enthusiasm for the maternity waiting home concept continued at the district level. Health managers, midwives, and even local governments sought funding from NGOs to build maternity waiting homes in their own districts. As a result, five additional facilities were built, which were modeled on the maternity waiting home concept (21). This adoption by district leaders marked a “tipping point” for maternity waiting homes in Timor-Leste.

Somehow it took on its own life. Part of it was during the course of 2005, something happened with this concept and it got its own dynamics, which I never fully understood. It was somehow, it went beyond the technical people and it started to be an idea that floated and there was far more interest in this concept than actually was meant to be … It just popped up out of nowhere that there will be waiting homes, waiting homes, waiting homes. – Advisor 2, UN agency, Dili

### Policy actors

Individual policy actors were highly influential in getting the maternity waiting home policy on the national agenda and socializing the idea to the districts. There was significant enthusiasm for the policy at many levels within the Ministry of Health, but one senior official stood out as the policy champion and promoted the idea at national workshops (15). The importance of this central figure was raised in most interviews. Participants identified him as the most significant actor influencing the change from the pilot approach to a country-wide strategy for every sub-district.

#### Values and Interests

As discussed above, a motivating factor in the expansion of maternity waiting homes was their highly visible and community-oriented nature. Thus, building maternity waiting homes in otherwise neglected areas was a way of demonstrating government action and promoting party loyalty.

It was the [senior decision-maker’s] personal agenda. It had something to do with the election perhaps. It was a good campaign strategy. – Manager, international NGO, Dili

#### External Actors and Influence

Although there was ownership and enthusiasm for maternity waiting homes evident at multiple levels within the Ministry of Health, there was growing caution voiced by representatives from international NGOs and UN agencies. Over time, maternity waiting homes became a much more contested policy in Timor-Leste.

So I don’t have that feeling that much right now, that it is going to be the one strategy. And I think pretty much from WHO, UNFPA, UNICEF, ourselves, I think everybody is aware, they are sceptical about whether or not that is the answer here. – Manager, international NGO, Dili.

Ultimately, it was senior decision-makers in the Ministry of Health who had the most influence over gaining support for the maternity waiting home policy. Representatives from NGOs and UN agencies sought to influence senior decision-makers to support other programs and priorities. Their mechanisms of influence included technical advice, writing proposals and policies, advocacy, and engaging in official dialog as well as informal discussions and relationship building.

Normally it will be the department who will act, it can be going both ways as well. I mean it can be that the Permanent Secretary, Planning, Vice-Minister, Minister, whoever, or an advisor, would have a strong view, but will ensure then that the department is on line for that. – Advisor 2, UN agency, Dili

A Ministry of Health official produced documents during an interview which showed that 60% of the 2006/2007 health budget was obtained from donors. Funding was an important avenue of influence in that donors could decide which strategies were funded. The Ministry of Health also influenced donors as they lobbied external agencies to fund their proposals and provide technical support to develop their policies.

You deal with the different organisations that come in to support then you have to make sure that they will support the objectives and the activities that you want. Not to create another vertical program because any organisation, they may have their policies, their role and function, and they are already committed to do something and you have to negotiate with our national leaders. If not then it can just increase the workload, you just start but never address the problem. – Department manager 1, Ministry of Health, Dili

Relationships between individuals were important mechanisms of influence and often transcended organizational affiliations. Very early on, staff within the Maternal and Child Health Department built alliances with a UNFPA advisor to progress the maternity waiting home policy. The success of these relationships depended on how long the advisor had spent in the country, complementary personalities, and common goals and values.

I think the challenge here in East Timor is also because there has been such an incredible flow of expats and advisors and stuff throughout the last five years, I think that there is a fairly high level of resistance on the part of Timorese to outsiders coming in and trying to take over or control the situation. So I think that there is probably a tendency amongst the Timorese here even to just want to deal with the people that they were familiar with. – Manager, international NGO, Dili

The relationships and alliances formed between technical advisors, NGO representatives, and Ministry of Health staff meant that common goals had greater influence and more chance of being supported. This is because NGOs and UN agencies had expertise and control of the funds and Ministry of Health staff had the legitimacy of the government. Advisors tended to have more influence at lower levels of government given management staff had less power, as well as the positioning of advisors as “experts.” Although advisors could be influential on their own, they still needed to work with the priorities of the government.

#### Decision-Making Power

Senior decision-makers heavily influenced the policy agenda through exercising power inherent in their positions. For example, after a piloting process had been agreed upon and formally written up as a strategy (15), a high-level decision-maker in the Ministry of Health sent a memorandum to managers in the Maternal and Child Health department saying maternity waiting homes were to be implemented in all 65 sub-districts in the country. This not only circumvented the original approach but also represented a radical departure from international guidelines which recommended that they are implemented close to functioning obstetric services (12).

The decision-making power evident in the top positions within the Ministry of Health was further demonstrated after the election of the new government in 2007. Senior decision-makers rapidly changed policy direction and the new focus became community level primary health care and sub-district maternity clinics (22). Thus, political priority for maternity waiting homes waned, which was aided by the skepticism shown by some UN agency representatives (15). The change in policy was a direct result of leadership changes and reflected the agenda of a senior decision-maker in the new Ministry of Health. It also coincided with the release of our in-country report which showed the maternity waiting homes that had been implemented were not improving access to birthing facilities for women in remote areas (23).

It’s normal for a new leadership to come in and have some ideas. He [a senior decision-maker] has been working in academia for a long time and he was probably exposed to the evidence, or the lack of, on maternity waiting homes. And he was just like ‘take it out’. – Project officer, international NGO, Dili

### The role of evidence

The first process evaluation of the maternity waiting home in Lospalos found it was rarely being used by women outside of the immediate town center (15). Despite these results, it was used as a success story by senior Ministry of Health officials. Therefore, rather than critically and objectively reviewing the available literature, evidence tended to be “constructed” by citing authoritative documents [i.e., Ref. (12, 15)] in order to support a given argument. The selective use of evidence was seen as the normal state of affairs in policy-making.

I think it just gets to be this kind of wave sort of thing where people hear about it and it’s like, ‘oh yeah that’s great’, without really looking at what does the literature say has been the history of these and how effective have they been. – Manager, international NGO, Dili

In addition to written documents, aspects of the local context were used to argue for the maternity waiting home policy (such as the high proportion of women living in remote mountainous areas), while other aspects of the context were ignored (such as women’s preference for home birth and the lack of adequate birthing facilities). Therefore, context and “logic” could be used to argue that research conducted elsewhere was not applicable locally.

If you have evidence outside it doesn’t mean necessarily that it will apply here … it doesn’t mean that because in Nepal [maternity waiting homes] didn’t work that here it cannot work. You know. Because it depends on the, it’s a cultural matter, there is a huge component, cultural component. – Advisor, UN agency, Dili

Department managers appeared to be very open to the use of evidence to inform policy and practice, but lacked decision-making power to influence the national policy agenda. For example, when the new government came into power and had different priorities, Department managers who were previously supportive of maternity waiting homes were highly compliant to new direction from their seniors.

So if you can provide [the department manager] the evidence and back it up and explain how it will work, she can be a really good advocate. And at the same time, you know, just that tradition of using evidence to inform practice is not always there. But that’s normal, it’s really normal. – Program officer, international NGO, Dili

With the maternity waiting home policy being continually challenged, first by technical advisors then by a change in leadership, our health system evaluation became part of the policy process. The fact that our research was local, relevant, and timely meant that the findings were taken up and used to justify a realignment of policy away from this approach.

It all depends on the evidence that you will present. If it’s good then we can support, but if it’s not good then I think we just forget, we have other things to do. – Department manager 1, Ministry of Health, Dili

It was fortuitous that the timing of this research coincided with the change in government in 2007. The new Minister for Health was a supervisor and collaborator in this study, and had intimate knowledge of the research and its findings. Because of his existing values, engagement with this and other research (24), as well as direct observation during field trips to the districts, his policy priorities were maternity clinics and community-based antenatal and postnatal services, enacted through the Servico Integrado Saude Communitaria (SISCa) strategy (25). The decision-making power inherent in top government positions illustrates the importance of involving these influential actors in research.

The ability to influence the policy agenda was also related to being able to present evidence in a persuasive way, as well as that evidence aligning with current political priorities. A further example was during the development of the Basic Services Package where the decision to stop providing home-birth services and support only facility-based delivery was, in part, the result of the evidence presented by an international consultant.

[The consultant] was not willing to compromise on the intermediary phase of moving from home births…I just know that he sent out the chapters for consultation and the three delays and everything that he outlines, it was really beautifully sketched to help people understand the problems. But the solution was a facility-based solution that he proposed, and people accepted that. – Program officer, international NGO, Dili

We had quite a detailed discussion both with the policy-makers, and with the technical department. And it was quite a system of going through the whole range of, I mean, one exercise was going through those Lancet, that series of articles both on child mortality and maternal mortality, and extracting the international experience from these, and cross checking to what extent they applied here or they didn’t apply here. That was one active exercise that was done during this process, as part of the BSP [Basic Services Package] process. – Advisor, UN agency, Dili

## Discussion

The most important aspects which led to the prominence of the maternity waiting home policy were (a) the political context which allowed the idea to be imported through international actors and gave Timorese control of health leadership after the referendum, (b) the characteristics of the policy in that it was a visible, non-controversial, and “logical” solution to the problem of access, and (c) the power of senior policy actors within the Ministry of Health who promoted the strategy that fit within their value system.

International policies are increasing in influence through processes of globalization, and the political context of a country can create windows of opportunity for those ideas to be transferred. This was illustrated in Timor-Leste through the influx of NGOs and UN agencies after the referendum and the prioritizing of maternal health as a national issue. The maternity waiting home concept spread through organizational structures linking technical advisors, such as those from UNFPA, with national Ministry of Health staff. Thus, global policy ideas could be communicated directly down a channel of influence. A country’s political context is important because it means newly established, poorly resourced or vulnerable states are more susceptible to the outside influence of organizations, individuals, and their values, as well as global policy trends. Steinberg and Baxter (26) have pointed out that community processes and structures create barriers to entry, and outsiders encounter less resistance entering systems that lack clear leadership.

The emergence of the Ministry of Health after Independence in 2002, and their relative coherence and strong leadership (27), resulted in ownership of the maternity waiting home policy and greater autonomy over which policies would be supported. This makes the maternity waiting home policy an interesting case study in that it received much more attention and support from the national and district level than was expected. This is in contrast to most other global strategies introduced in low-income countries, for example, HIV policy (28, 29), the Safe Motherhood Initiative (30), and DOTS in tuberculosis treatment (31).

With the proliferation of NGOs and UN agencies in weak states it is widely held that global policies and donors have significant influence over national policy frameworks. Our findings from this case study, however, support Shiffman (32) conclusions that efforts by national leaders were more important than that of international donors in predicting whether maternal mortality made it onto the policy agenda. Similarly, Behague et al. (3) (p. 1544) also found “a powerful group of national elite control research and policy agendas.” They argue that these national elites have a strong vested interest in global undertakings and tend to be removed from the local realities found in their countries.

Cobb et al. (33) have proposed a “mobilization model” of policy which is worth revisiting because it closely reflects the policy process surrounding maternity waiting homes in Timor-Leste as well as that described by Shiffman (32) and Behague et al. (3). In this model, government decision-makers place issues on the agenda, then promote the idea and gather support for implementation because they lack institutional and financial resources. This model of policy is most often found in hierarchical societies and in contexts where there is social distance between political elites and the public (33). Similarly, Mills (34) argues that rather than policy reflecting the needs of the people, it most often represents the interests of the most powerful individuals within society and the broader ideologies they support. Thus, it appears the models for policy developed decades ago in high-income countries (19, 33, 34) are still applicable in a range of diverse contexts today, including low-income and post-conflict settings. The EBP movement is therefore an important shift.

The way in which evidence was used in advocating for maternity waiting homes in Timor-Leste reflects Howlett and Ramesh (35) (p. 122) experience in that “symbols and statistics, both real and fabricated, are used to back up one’s preferred understanding of the causes of the problem.” Rather than EBP, it appears the “solution” of maternity waiting homes became joined to the “problem” of access (2). In addition to drawing on documented case studies of maternity waiting homes (12, 15), advocates cited certain aspects of the local context in order to justify the policy (difficulties with transport and remoteness) while simultaneously ignoring other underlying requirements [lack of quality birthing facilities, electricity, running water and emergency obstetric care, see, for example, Ref. (15, 23, 36)].

This use of evidence is consistent with the literature, which suggests policies are legitimated rather than informed by evidence (3). The problem with this approach is that it limits the use of research for context-specific problem solving. Behague et al. (3) (p. 1542) studied maternal and neonatal health policy in five developing countries and found “new ‘fad-like’ evidence-based policies tend to be proposed and adopted with such enthusiasm that they undermine a comprehensive approach, and are used to replace, rather than complement, previous endorsed policies.” In their review of theory in health policy change, de Leeuw et al. (18) found policy is grounded in value-based rather than evidence-based ontologies and tends to address a tangible yet insignificant element of the complexity of the real problem.

So what does this mean for the role of evidence in policy-making, and the EBP movement in general? We argue that it is important to recognize that politics surrounding evidence is inevitable (37) and that policy-making will never be free of bias (nor will research, which is why it is so critical to be reflexive and open about our own values). The value of rigorous, context-specific research is that advocacy groups and policy champions have the necessary data to back their case or indeed to refute it. Our research was taken up by policy-makers because it addressed an existing policy problem, was timely and credible. Credibility was generated not only through the use of rigorous scientific methods, but by being affiliated with an independent organization and connected to high-level decision-makers. Involving policy-makers from the outset of the research was an important factor in the latter.

Given the extensive examples in the literature which illustrate how evidence is used to legitimate policy (2, 3, 18, 35), the type of research that is conducted and for whom is a critical question. We argue for a national research agenda that incorporates the views of stakeholders who are “seldom heard” rather than developing research priorities based only on a review of existing literature (38) and the priorities of the elite. To this end, rural women need to be included in developing and conducting maternal health research. The Timor-Leste Cabinet for Health Research and Development (CHR&D) was established in 2010 to facilitate and promote health research. We argue that it is critical that the CHR&D incorporate a rural women’s research and policy agenda to understand what mix of facility, community, and home-based antenatal, birthing, and postnatal services work best for women and families and for reducing morbidity and mortality, and to feed this research into policy improvements.

## Conclusion

This research demonstrates the importance of political context, policy characteristics, and the power of national elites in determining which policies are supported. We found high profile actors within the Ministry of Health supported policies in their bureaucratic interests and broader values. External actors, however, had much less influence over the maternity waiting home policy than expected in this context. The characteristics of the policy meant maternity waiting homes were an attractive option because they were visible and non-controversial, provided a logical solution to a priority problem, and were consistent with the dominant ideology of facility-based births. The people most affected by the policy, particularly pregnant and/or rural women, had very little influence over the maternity waiting home agenda, or maternal health policy in general.

Our study of the policy process showed that research was used to both inform new policy directions and support pre-defined policy agendas. This confirms the finding that policy-making is a symbolic sense-making activity rather than a purely methodological enterprise (18). Thus, the potential impact of research on policy lies in its timeliness and relevance and the connection between researchers and policy-makers. The next steps in the pursuit of EBP entail not only working with policy-makers to set the research agenda but also incorporating the views of those “seldom heard” in order to conduct research that adequately addresses the complexity of real-world problems. The effect of these participatory models should be evaluated.

## Author Contributions

KW led this research as part of her Doctor of Philosophy and wrote this manuscript. LB, PK, and NM, in their capacity as supervisors, provided input into the research design, interpretation of data, critically revised earlier drafts, and approved the final manuscript.

## Conflict of Interest Statement

Co-author Nelson Martins became the Minister for Health during the time this research was conducted (2007), and remained the Minister for Health until 2012. We have outlined in detail how this may have impacted the research, see Materials and Methods section of the manuscript.
